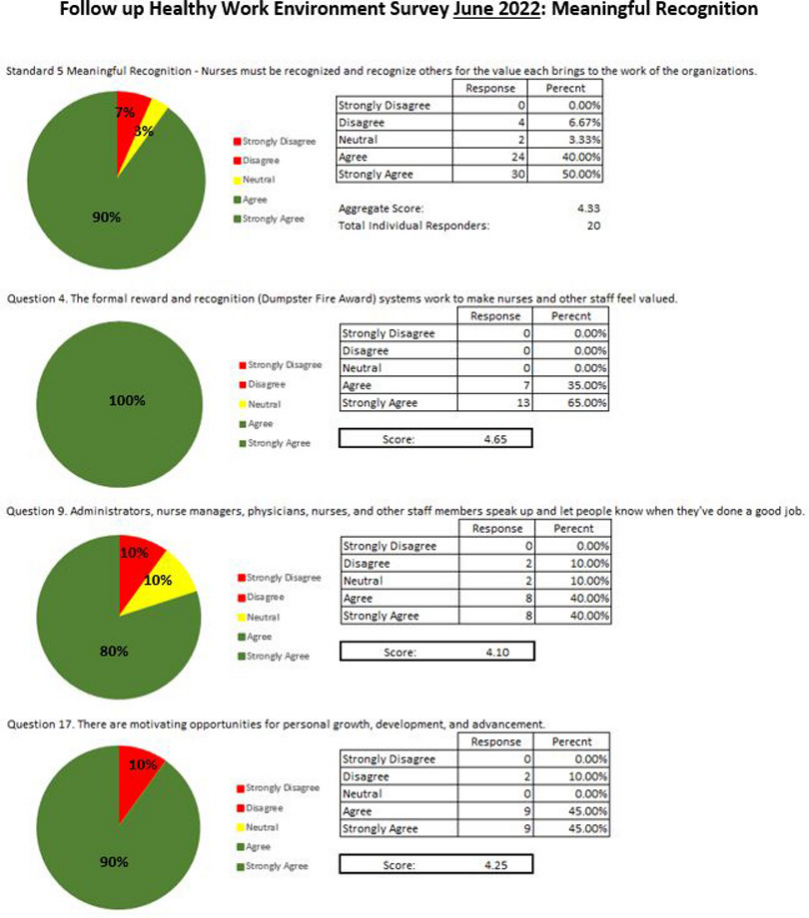# 308 Meaningful Recognition Makes a Difference: Turning Dumpster Fires into Dumpster Gold

**DOI:** 10.1093/jbcr/irad045.283

**Published:** 2023-08-29

**Authors:** Joseph D Knapp

**Affiliations:** Warden Burn Center at Orlando Regional Medical Center, Orlando, Florida

## Abstract

**Introduction:**

Burn/Trauma Critical Care Nursing requires a team effort to achieve positive outcomes. Meaningful Recognition is one standard of a Healthy Work Environment and a challenge to provide consistently in today’s climate. In 2021 we conducted a HWE assessment survey and under the category of MR our aggregate score was 2.94 or “Needs Improvement.” The phrase “Dumpster Fire” has been used to describe the most challenging clinical shifts in nursing within our unit. A DF shift on our unit has been undefined due to the complexity of the care provided to patients and without any formal method specific to our unit in acknowledging the staff that care for the most critically ill patients. We defined a DF to create a specific award in which staff nominate each other for the skill demonstrated to provide excellent care and the good outcomes. We implemented the award as a post survey action item with a goal to achieve an aggregate score of >=4.0 or “Good,” on a follow up survey on MR in June of 2022.

**Methods:**

MR is one of six standards presented by the AACN (HWE) assessment tool to aid in developing plans to increase retention and satisfaction among staff at the bedside. Our intervention period began in April 2021 and ended June 2022. During this time staff, allied health personnel, physicians, and unit leaders can nominate others for a DFA. The story of the nominees and the teamwork necessary are shared at weekly staff huddles. The nominees would also receive a dumpster fire enamel pin provided by unit leadership and have their picture taken for the "Wall of Flame" on the unit and our social media page. by sharing the story with the team which was presented to the nominee(s) during shift change huddles.

The scoring guidelines are as follows: 1.00-2.99 = Needs Improvement, 3.00-3.99 = Good, 4.00-5.00 = Excellent

**Results:**

Standard 5 Meaningful Recognition Nurses must be recognized and recognize others for the value each brings to the work of the organization.

2021 Aggregate Score 2.94

2022 Aggregate Score 4.37

Question 4 The formal reward and recognition systems work to make nurses and other staff feel valued.

2021 score 2.45

2022 score 4.62

Question 9 Administrators, nurse managers, physicians, nurses, and other staff members speak up and let people know when they have done a good job.

2021 score 3.64

2022 score 4.17

Question 17 There are motivating opportunities for personal growth, development, and advancement.

2021 score 2.73

2022 score 4.31

**Conclusions:**

Based on the results of the post intervention survey there was a significant increase in the individual and aggregate scores for MR. This data demonstrates that a unique and meaningful method of recognition can impact the perceptions of staff of a HWE within a busy level I trauma center’s Trauma Intensive Care Unit.

**Applicability of Research to Practice:**

Meaningful Recognition is an important part of a Healthy Work Environment and a fully implemented HWE initiative can help mitigate staff losses and help build resilience among nursing staff and the treatment team members.